# Study protocol: longitudinal study of the transition of young people with complex health needs from child to adult health services

**DOI:** 10.1186/1471-2458-13-675

**Published:** 2013-07-23

**Authors:** Allan F Colver, Hannah Merrick, Mark Deverill, Ann Le Couteur, Jeremy Parr, Mark S Pearce, Tim Rapley, Luke Vale, Rose Watson, Helen McConachie

**Affiliations:** 1 Institute of Health and Society, Newcastle University, Sir James Spence Institute, Royal Victoria Infirmary, Newcastle upon Tyne NE1 4LP, UK; 2 Institute of Neuroscience, Newcastle University, Sir James Spence Institute, Royal Victoria Infirmary, Newcastle upon Tyne NE1 4LP, UK

**Keywords:** Transition, Adolescence, Chronic illness, Disability, Evaluation, Protocol

## Abstract

**Background:**

Young people with complex health needs have impairments that can limit their ability to carry out day-to-day activities. As well as coping with other developmental transitions, these young people must negotiate the transfer of their clinical care from child to adult services. The process of transition may not be smooth and both health and social outcomes may suffer.

Increasingly, policy-makers have recognised the need to ensure a smoother transition between children’s and adult services, with processes that are holistic, individualised, and person-centred; however, there is little outcome data to support proposed models of care. This study aims to identify the features of transitional care that are potentially effective and efficient for young people with complex health needs making their transition.

**Methods/Design:**

Longitudinal cohort study. 450 young people aged 14 years to 18 years 11 months (with autism spectrum disorder and an additional mental health problem, cerebral palsy or diabetes) will be followed through their transition from child to adult services and will contribute data at baseline, 12, 24 and 36 months. We will collect data on: health and wellbeing outcomes (participation, quality of life, satisfaction with services, generic health status (EQ-5D-Y) and condition specific measure of disease control or management); exposure to proposed beneficial features of services (such as having a key worker, appropriate involvement of parents); socio-economic characteristics of the sample; use of condition-related health and personal social services; preferences for the characteristics of transitional care.

We will us regression techniques to explore how outcomes vary by exposure to service features and by characteristics of the young people. These data will populate a decision-analytic model comparing the costs and benefits of potential alternative ways of organising transition services.

In order to better understand mechanisms and aid interpretation, we will undertake qualitative work with 15 young people, including interviews, non-participant observation and diary collection.

**Discussion:**

This study will evaluate the effect of service components of transitional care, rather than evaluation of specific models that may be unsustainable or not generalisable. It has been developed in response to numerous national and international calls for such evaluation.

## Background

Young people with complex health needs (CHN) are those with a physical, mental or health impairment that has the potential for a substantial and long-term adverse effect on their ability to carry out normal day-to-day activities [1]. Therefore, as well as coping with other developmental transitions [2], young people with CHN have to negotiate the transfer of clinical care from child to adult services. Many young people with CHN have poor health and social outcomes following transition [3,4], regardless of the nature of the condition [5-9].

The importance of transitional care to the wellbeing of young people with CHN is recognised in recent UK health policy [10]. Recommendation 32 of the 2010 Kennedy report [11] stated, ‘Ensuring a smooth transition between children’s and adult services should be a priority for local commissioners’. The 2011 Green Paper on special educational needs and disability [12] highlighted the importance of good transitional health care and proposed an annual review by their general practitioner for every young person with disability. However, while much research and policy agree that transition processes should be holistic, individualised, and person-centred, there is a paucity of outcome data to support proposed models of care.

Internationally, similar problems and policy responses have been identified. The American Academy of Pediatricians reported that after a decade of effort, health transition support as a basic standard of care had not been implemented [13]. The Canadian Pediatric Society issued two position statements stating paediatricians should be flexible and highlighted the importance of considering the child’s developmental age when planning transition to adult services [14,15]. Research in the Netherlands has reported studies of adolescents’ and their parents’ experiences of transition [16,17], and more recently [18] evaluation of transition programmes available and the effect of transition to adult services on young people.

In adolescence, the challenges for young people include seeking the kind of education or training they want, thinking about when and how to live independently, and making romantic relationships. Therefore, the primary policy initiatives about transition have focused on social services and education. For health services the complexity of transition, due to its multidisciplinary, interagency nature, is a challenge, even when they have the strategic will and resources to develop services. User-friendly and effective health protocols will not be efficient if services in other agencies are absent or inadequate. The UK Government’s proposal that more health service commissioning should be by Local Government Authorities is a significant opportunity to ensure that services for transitional care are not commissioned in isolation from services in other sectors.

Over the last decade, there has also been a new understanding of adolescent brain development and its association with adolescent behaviour [19]. Particular regions of the brain, such as the prefrontal cortices, continue to mature into the middle twenties. These areas support executive functioning, time planning, and inhibition of inappropriate emotional impulses. It is therefore not surprising that many young people with CHNs find it difficult to negotiate the transition to adult services. Approaches to improving transition must be set in a developmental context.

### Need for research in this area, and the rationale for the lines of research we adopt

A scoping review of transition models found limited literature evaluating health services for young people with diabetes or cerebral palsy, and none for young people with autism spectrum disorder (ASD) [20]. Most publications described either new services or time-limited pilot studies with little evaluation or evidence of sustainability. This confirmed work by the McMaster Group which found little data on which to decide if introduction of dedicated services would improve outcomes [21].

There are some examples of promising interventions in health services. The Diabetes Navigator study showed improvement in clinic attendance, although there was no reduction in hospital admissions [22]. A UK study of a dedicated programme for transition in juvenile arthritis showed short-term benefit in patient and parent satisfaction, disease knowledge and health-related quality of life [23]. A UK study of an integrated paediatric/adult service for chronic kidney disease showed improved medication adherence and engagement with healthcare providers, judged by reduced transplant failure rates [24]. Crowley et al’s systematic review [25] found most interventions use strategies of either patient education, staffing (joint clinics, named transition co-ordinator) or service delivery (separate young adult clinics). Transition programmes for patients with diabetes were the only ones to show significant improvements in outcomes. Such studies provide preliminary evidence for beneficial features of services that should now be tested in other conditions and settings [25].

A report from the York Social Policy Unit found little research on transition of young people with complex physical impairments [26]. While it found some progress in policy developments, it found no evidence about whether policy influences health and social outcomes; a need that has been stressed in recent government papers [27].

There is still less research on transition of young people with neurodevelopmental disorders, such as ASD or attention deficit hyperactivity disorder, although attempts are now being made to define service needs [28]. The UK Autism Act [29], accompanied by statutory guidance ‘Fulfilling and Rewarding Lives’, specifically requires Local Authorities to plan transition services for people with ASD [30], Disparities in transition planning for young people with ASD in comparison to young people with other special healthcare needs have also been reported in the United States [31,32].

There have been few studies on cost-effectiveness of transitional care, even though the personal and social costs of unsuccessful transition may be substantial [33]. Unsuccessful transition can lead to reduced appropriate contact with health services. This may result in negative health outcomes such as increased emergency admissions, disease complications, and long-term health and social problems, which impose still greater costs on health services [34-36].

In our study, we focus on young people aged 14 to 21 years. During this period, young people’s status changes with respect to consent, education and independence; and service provision and commissioning change from paediatric to adult health services. We will examine three ‘exemplar’ conditions that give rise to complex health needs: autism spectrum disorder and an additional mental health problem, cerebral palsy or diabetes. The young people should have intellectual ability in the average range (i.e. no significant learning disability); in the UK there are no multi-agency models of transition for such young people [37], unlike young people with intellectual disability who are supported by learning disability teams. Those with intellectual ability in the average range should have sufficient personal autonomy to contribute to negotiations about their own health care during transition.

### Purpose and aims of the overall transition research programme

The research programme aims to promote the quality of life and health of young people with CHN by generating evidence to enable NHS Commissioners and Trusts to facilitate successful transition of young people from child to adult health care, thereby improving health and social outcomes. The programme has three overall aims:

1) To work with young people with CHN to determine what successful transition means to them and what is important to them in their transitional care.

2) To identify features of transitional care that are effective and efficient.

3) To determine how transition care should be organised, provided and commissioned.

### Aim and objectives of the longitudinal study – the subject of this protocol

The aim is to identify features of transitional care that are effective and efficient (Aim 2 above):

This longitudinal study has four objectives:

i To examine whether proposed beneficial features of services contribute to positive outcomes for young people with diabetes, cerebral palsy and autism.

ii To understand how service features contribute to positive outcomes.

iii To determine resource use, costs and strength of preferences about care delivered.

iv To undertake a cost-consequence analysis.

## Methods/Design

The primary design is a longitudinal study capturing quantitative data on health outcomes, exposure to features of services identified from the research literature as potentially beneficial, socio-economic characteristics of the sample, and data to allow health economic analysis. We will visit 450 young people with complex health needs annually on four occasions (follow-up over three years) through their transition from child services to adult services. In order to further understand and interpret the findings of the longitudinal study, we will undertake qualitative work with fifteen of these young people, including interviews, non-participant observation and diary collection.

We will exclude young people with significant learning difficulties because the aim of the study is to capture young people’s opinions about their lives and transition services, without the need to use proxy reporting.

### Participants

Participants will be aged 14 years to 18 years 11 months at the beginning of the study. We will draw them from young people with one of three long-term conditions: diabetes for chronic illness; cerebral palsy for complex physical impairment; autism spectrum disorder with mental health difficulties for neurodevelopmental disorder. Their transfer of health care to adult services should not have started but be likely to begin in the coming year. The young people must have abilities in the average range of intelligence. We will seek 150 participants for each exemplar condition. One parent/carer for young person will also be invited to participate.

### Recruitment

We will recruit young people with diabetes from outpatient clinics in four hospitals across England, selected because they were in the process of developing transition services. We will recruit those with autism from four child and adolescent mental health services across England. We will recruit those with cerebral palsy from two regional population registers in northern England and Northern Ireland.

Once a young person has consented to take part in the study, the research assistant will tell them about the qualitative study.

### Data collected and instruments used

1 Baseline socioeconomic data. For the parent/carer we will collect data on ethnicity, household income, educational level and occupation. For the young person we will collect data on ethnicity, work, educational status, where the young person is living and with whom.

2 Later socio-economic data. For the young person we will update the above data at each visit.

3 Outcomes. We will capture the following at each visit:

• Participation: The Rotterdam Transition Profile [38] includes nine items measuring education and employment, finances, housing, transportation, romantic relationships, leisure, health care needs, services and aids, and specialist services for your condition. Participants select the statement that best describes their current situation. Each statement represents one of three phases; Phase 1 – the young person is still dependent on their care givers, phase 2 – the young person is learning skills to be independent but is not independent yet, and phase 3 – the young person is independent (although may still require support). We found the measure to be acceptable across conditions in pilot studies conducted by the Programme team in 2010.

• Subjective wellbeing: Warwick-Edinburgh Mental Well-being Scale [39] is a 14-item scale validated for teenagers and young adults. All items are measured on a 5-point scale from “none of the time” to “all of the time”.

• Patient and carer experience of services: Mind the Gap [40] consists of 22 items (young person version) and 27 items (parent version). Participants are first asked to answer the questions thinking about the idea of their ‘best’ healthcare, and then again about their current healthcare experiences. Responses are on a 7-point Likert scale anchored by ‘strongly disagree’ at 1 and ‘strongly agree’ at 7. Satisfaction with each item is the ‘gap score’ between their ‘best’ and ‘current’ score.

• Health Outcome: EQ-5D-Y [41] has five dimensions on: mobility, looking after self, usual activities, pain and discomfort, and mood. Each of the dimensions has three levels of perceived problems: 1: no problem, 2: some problems, and 3: extreme problems. A unique health state is defined by combining one level from each of the five dimensions (e.g. 12132).

• Condition specific measures of disease control or management, as shown in Table 1.

**Table 1:** Condition-specific measures

**Instrument**	**Completed by**	**Captures**	**Domains**	**Measured at**
**ASD**				
Social responsiveness scale [42]	Parent	Severity of ASD characteristics	Awareness	Baseline
			Cognition	
			Communication	
			Motivation	
			Mannerisms	
Strengths and difficulties questionnaire [43,44]	Parent and young person	Emotional and behavioural problems	Emotional symptoms	Baseline
			Conduct problems	
			Hyperactivity	
			Peer problems	
			Pro-social behaviour	
Hospital anxiety and depression scale [45]	Young person	Severity of mental health problems	Anxiety Depression	All contacts
Children’s global assessment scale [46]	Clinician	Functional impairment		Baseline
**Cerebral palsy**				
Severity of impairments	Research assistant	Impairments	Type of cerebral palsy, gross motor function, upper limb function, intelligence, hearing, vision, communication, feeding, seizures	Baseline
Unmet health needs	Parent and young person			All contacts
**Diabetes**				
	Research assistant	Diabetes control	Age at diagnosis	Baseline
	Research assistant		HbA1c averaged over last year, number of admissions for ketoacidosis over last year, result of annual eye examination	All contacts

4 Proposed Beneficial features of services

Table 2 shows the nine service features to which we shall establish whether the young person is exposed. In Table 2 are references to guidance that recommends the feature and references to preliminary evidence that the feature is beneficial.

**Table 2:** Proposed beneficial features

**Feature**	**Guidance**	**Preliminary evidence**	**Assessment by young person**	**Assessment by parent/carer**	**Assessment by health care provider**
**C** Age banded clinic(s)	[47]	[48,49]	Yes/No		
			If yes		
			Adolescent clinic		
			Joint clinic (shared staff)		
			Young adult clinic		
**A **Meeting the adult team before transfer of care	[47]	[36,50-54]	Yes/No		
			If yes		
			Joint clinic (shared staff)		
			Meet for introduction		
**S **Active promotion of health self-efficacy	[55-59]	[60-70]	Have you received enough help to increase your confidence in managing your condition?		Do you have a policy/protocol for promoting health self-efficacy?
			Scale 1-3		
			Enough help		
			Not enough help		
			Did not need help		
**TP **A written transition plan	[58,59]	[23,63,71]	Do you have a written plan that includes the plan for health care?		Do you aim for there to be a written transfer plan for every young person? Yes/No
			Yes/No		
**P **Parental involvement.	[58,59]	[50,60,72,73]	P(a) Has your parent/carer been consulted about and involved in your care.	P(c) Have been consulted about and involved in your child’s care?	Do you have a parental engagement policy?
Keeping parents engaged in their child’s care but with different responsibilities. Whilst the young person must separate from parents, total independence is not the aim. All young people will seek advice from parents. Some with CHN remain more dependent on parents than others.					
			Yes/No		
			P(b) Is this the right amount, too much or too little?	Yes/No	
				P(d) Is this the right amount, too much or too little?	
**K **Key worker.	[10,55,58,59,74-76]	[22,37,77-80]	Have you got or had a key worker who works with you?		Do you have a policy for ensuring every young person has a key worker?
A single person the young person can approach to sort out any problems around health care?					
			Yes/No		
			Who is it?		
Does not have to be formally allocated.					Yes/No
Does not have to be a health provider but must be able to influence health services.					
**M** Services co-ordinator working at managerial level	[55]	[34,49]			Is there are co-ordinator of transition and transition clinics? Yes/No
**T** Convenient coordinated care	[59]	[48]	In the clinics you attend, can you see a variety of professionals such as doctor, psychologist, therapist on the same day or place?		Do your follow up clinics offer a variety of professionals such as doctor, psychologist, therapist, dietician?
			Yes/No/Not applicable		Yes/No
**H **Formal training, relevant to health condition, in wider life skills - education, relationships, health maintenance etc. Holistic approach with contacting of other agencies and if appropriate sign posting young person to other agencies as appropriate	[58]	[38,81]	Yes/No		Do you provide life skills modules or ensure they are available from other providers?
					Yes/No

*YP *Young people.

We will collect information on exposure to these features at visits two, three and four. We will use two prompts to help the young people remember what has happened during their contacts with health services in the previous year: a) an appointments log kept by the young person of healthcare contacts; b) researchers (with consent) will access the young person’s health records before each visit.

We shall also ascertain from the health service provider whether it thinks it provides such features.

5 Use of services and costs

We will collect these data at visits two, three and four. The two prompts outlined in the paragraph above will aid recall of utilisation of services during the previous twelve months, as a basis for obtaining service costs. We will also ask participants and those accompanying them about their usual mode of transport, distance, how long on average they spent travelling to and from and attending each contact, and what they would otherwise have been doing.

6 Discrete Choice Experiment (DCE)

At the third visit, we will use a DCE to determine the relative importance (utility) to young people with CHN of different service configurations. A DCE is an attribute-based method of benefit measurement used increasingly in health care [82]. It elicits preferences in situations where health and healthcare can be characterised by a number of attributes. In a DCE a set of choices, that vary with respect to the level of the attribute, is presented to individuals. We will base the attributes and levels for the DCE in part upon the finding of a Q-sort study conducted earlier in the Transition Programme.

### Qualitative study: young People’s experience of transition

We will explore young people’s experience of healthcare transition services by asking all young people enrolled into the longitudinal study if they would like to take part in this qualitative component, if chosen. We will explore how their experiences of transition health services evolve over time through conducting qualitative interviews at two time points. Five young people from each exemplar condition (n = 15 in total) will be followed over a two to three year period. Location, age and phase of transition will guide the purposive sampling.

The interviews will focus on the young person’s experiences of, understandings of, views about and aspirations for their healthcare transition. We will ask the young people to record their personal transition journey by suggesting to them some ways to record their experiences - written journal, photographs, audio diary, video diary, blog - but we will be open to suggestions from them. Each young person will nominate a ‘significant other’ (such as parent, sibling or partner) and a key healthcare professional involved in his or her transition. With the consent of the young people and relevant clinician(s) and anyone accompanying the young person, we will also observe their clinical encounters with a range of healthcare providers (n = 1-4 observations of encounters per young person; n = 15-60 observations in total).

### Training of research assistants

#### ***Quantitative study***

We will appoint research assistants (RA) in each region for each exemplar condition. They will attend training workshops in the co-ordinating centre, Newcastle. Before recruitment begins, the first workshop will enable them to learn about the study and its methods, to ensure consistency across sites and to promote engagement with and enthusiasm for the study. The training will cover: the rationale for the study; the National Health Service regulatory framework for the conduct of research studies; how to engage young people and take informed consent; and how to administer the questionnaires. The RAs will also receive information about diabetes, cerebral palsy and autism and receive additional individualised training about issues to consider when approaching young people with one of those conditions. After each RA has completed about four visits there will be a second workshop to address any difficulties encountered, and explore strategies for maintaining contact with those recruited. Before the second year, a further training workshop will prepare the RAs for maximising retention, obtaining information from medical records, and for administration of the additional questionnaires.

The visits to the young person by the RA will usually take place in their home or alternative venue to suit the young person and their parents. The questionnaires are self-completed but the RA will explain them, answer queries that arise and, for young people with motor or communication difficulties, assist with their administration.

#### ***Qualitative study***

Another RA will undertake the qualitative work. She will receive the same training as those involved in the quantitative study. Additionally, she will receive training on how to conduct interviews and observations. She will attend regular meeting to explore any difficulties encountered.

### Retention of participants

Over the three years, some young people will leave home and receive healthcare elsewhere. At each visit, we will check contact details to ensure these are as up-to-date as possible to minimise the risk of loss to follow-up due to being unable to contact family. We will offer young people a £5 voucher as an incentive to continue their participation (visits two, three and four), and they will receive a certificate recognising their contribution to the research after each visit. The RAs will offer continuity to the young people and their families over the study period by keeping in regular contact via text messages, emails, and telephone calls.

### Data management

#### ***Quantitative data***

The research assistants will send photocopies of completed questionnaires to Newcastle University where we will enter the data into an Access database. Data entry and validation will be a continuous process, with a proportion of double data entry, so that we will identify and address problems immediately, rather than problems only becoming apparent on completion. We will download securely into the study database any data entered electronically by the young person via Survey Monkey. We will anonymise and store securely at each site information accessed from health records.

#### ***Qualitative data***

We will audio-record and transcribe all interviews. We will transcribe audio or video diaries and video clips produced by the young people. We will assimilate local documentary materials, including written diary extracts or photographs generated by young people. Non-participant observation in clinical settings will involve the production of contemporaneous field notes. When acceptable to the participants, we will audio-record some consultations to gain an objective record of them. We will edit all these transcriptions, documents, videos, photographs field notes and consultation notes to ensure anonymity of respondents.

### Project management

Responsibility for delivering all objectives of the Programme, which includes the work in this protocol, lies with the Programme Management Board. It consists of all fourteen co-applicants for the grant and is chaired by the Chief Investigator, AC. HMcC leads on the quantitative data collection, TR leads on the qualitative work and MD leads on the health economic work described in this protocol.

An external advisory group will meet about once a year, comprising two chief executives of health service Trusts, two young people with complex health needs, two people representing the voluntary sector, a general practitioner, three academics in sociology, rehabilitation science, nursing studies, and a representative of the funder – the National Institute for Health Research.

### Ethics

The study received ethics approval from Newcastle and North Tyneside 1 Research Ethics Committee. Numbers 12/NE/0059 and 12/NE/0284. All young people will provide signed consent to join the study. For young people under sixteen years of age, a parent will also provide signed consent for their child to join the study.

### Statistical analysis

We will examine the pattern of missing data and if appropriate use imputation, the exact method depending on the nature of missing data. We will assess for potential bias due to non-response by ensuring that baseline characteristics concerning age, gender, socioeconomic status as estimated from postcode data and disease severity/control are collected about non-responders. We will assess whether there are significant differences between those who do and do not take part by using tests such as chi-squared, t or Mann Whitney, depending on the nature of the data being assessed. There is likely to be less than 100% retention for subsequent visits and we will use the above methods to adjust further for potential bias.

We will use both individual outcomes and statistically determined combinations to characterise a ‘good outcome’. Descriptive statistics will be reported with initial tests for differences and associations, for example between the different clinical groups, assessed using chi-squared, t and Kruskal Wallis tests and ANOVA as appropriate.

Adjusted modelling will follow a standard regression framework with appropriate link functions. Differences between time points will be used as the outcome measure, adjusted for time between measurements as a covariate. For continuous outcome measures, we will use linear regression models with transformations if required to explore associations between groupings of proposed beneficial features, and other explanatory measures and outcomes. We will use ordered logistic regression models to analyse ordinal outcomes. We will develop adjusted regression models, including model of associations between explanatory variables and allowing adjustment for potential confounders and assessment of potential interactions, to explore pathways to outcomes. While initial analyses will include all data, regardless of clinical group, stratified analyses will explore whether different pathways are likely for the different clinical groups.

Observations are likely to cluster within Trusts and clinics; multi-level modelling will address this. We will use robust standard errors to take into account clustering of patients in any non-multi-level models.

#### ***Statistical power***

We aim to have 450 young people (150 in each of the three clinical groups) and estimate that 80% of the young people will be seen on all four occasions, that is at least 360 in total or 120 for each clinical group. Given this, as an example of our likely statistical power, we estimate that we will have 80% statistical power at the 5% significance level to detect the following at the 5% significance level:

i) a difference in proportion on the Rotterdam Transition Profile[38], illustrated by the rehabilitation/health care domain, of 0.13. For a subgroup of 120, the detectable difference would be 0.25.

ii) a within subject change in proportion on the Rotterdam Transition Profile (rehabilitation/health care domain) of at least 0.07. For a subgroup of 120, the detectable difference would be at least 0.25.

As no appropriate data were available on likely changes or differences, we chose to power the study based on the initial measurements. Using repeated measures methods will be more powerful. Clustering of data, for example within NHS Trusts, is likely to lead to wider confidence intervals around effect estimates in the regression models and with it less statistical power. However, this depends on the impact of clustering on the associations between exposure and outcome measures, which is unknown at this stage.

#### ***Estimation of costs***

We will combine resource use data with unit costs from public sources [83] and study specific estimates. We will report total costs for each year and for the whole follow-up period, and separately for the NHS and patients. We will discount costs at recommended rates for the evaluation of health and social care interventions. We will analyse cost data using regression techniques, appropriate to the likely skewed data, to explore the determinants of change in costs over time and of total costs.

#### ***Discrete Choice Experiment (DCE)***

We will use logistic regression techniques to analyse the data gathered from the DCE at visit three [82,84]. This will allow consideration of the relative importance of the individual characteristics of the service. If a price proxy is included then this will allow the estimation of willingness to pay. We will use these data in a subsequent economic evaluation model along with the findings of the economic data to compare the services. In effect, we will attach monetary values to attributes and levels of alternative ways of organising transition services. Furthermore, we will express the outputs of the economic evaluation model in terms of these attributes and therefore we will have estimated net benefits (in monetary values) for each strategy considered.

#### ***Cost consequence analysis***

The aim is to assess the relative efficiency of illustrative models of transition, using cost-consequence analysis. We will ask the NHS Trusts in the study to cost the resources required to deliver an illustrative set of transition services that vary by the levels they attain of the proposed beneficial features. Analyses from the longitudinal study data will allow us to estimate the cost consequences and benefits predicted for a given service configuration. We will use results from the DCE to aggregate relevant outcomes into a single measure of the demand for specific ways of organising/supplying transition services.

#### ***Qualitative work***

Anonymised transcripts, field notes and other documents will form the data for formal analysis. Ethno-methodology will theoretically-inform the analysis. We will conduct all analyses according to the standard procedures of rigorous qualitative analysis [85]. We will use procedures from first-generation grounded theory (coding, constant comparison, memoing) [86], from analytic induction (deviant case analysis) [87] and from constructionist grounded theory (mapping) [88]. We will undertake independent coding and cross checking and a proportion of data will be analysed collectively in ‘data clinics’ where the research team share and exchange interpretations of key issues emerging from the data.

## Discussion

Those committed to improving transition processes for young people with complex health needs are moving from descriptions of the problem and suggestions for improvement to actual evaluation of services and conduct of trials. Our five year Programme of research is part of such endeavour and within our Programme there is a planned longitudinal observational study that we have described in this paper.

Whilst the overall Programme has research components unique to the UK context, such as how transition services should be commissioned, the longitudinal study formalises evaluation that has been called for by reports from many countries, namely pre-specification of outcomes and examination of the influence of proposed beneficial features of transition services.

Tsybina et al [89] published their study protocol (LETS) for an ‘outcome evaluation’ of a longitudinal study of transition and it is instructive to compare their proposal with ours. LETS evaluates a specific programme of rehabilitation for young people with cerebral palsy or later acquired brain injury and compares its outcomes with a retrospective cohort managed by the same centre before the specific programme was introduced. The evaluation includes many young people with severe intellectual impairment and therefore only proxy outcome measurement can be obtained for them. Like our study, the main arm is prospective, following young people as they proceed through transition. It measures similar outcomes, albeit using different instruments, and it includes a qualitative component to assist interpretation of findings. The main outcome is ‘continuity of care’, which is a process measure rather than a health outcome for young people.

In our study, subjective well-being, participation and health status are the primary outcomes. We have piloted in the study population the instruments we shall use to capture these outcomes and the instruments are appropriate for use in all three exemplar conditions. The youngest participants will be aged 14 years at the beginning of the study and the eldest will be aged 22 years by the end of the study. Thus, we can investigate outcomes at all stages of the transition process, including preparation for transition, transfer to adult services and post-transition in adult services. This design also allows for the variability in the age at which young people in each condition begin the transition to adult services.

While evaluation of transition pathways and services is beginning to be established in diabetes care [90,91], there is still little evaluation or progress for chronic physical impairment or neurodevelopmental disorders. Because we cover three broad groups, we think that findings common to all of them will be likely to be generalisable to all young people with complex health needs. The participants will also come from several different services within the UK, giving more reason to believe that findings can be generalised to all young people with CHN accessing different services.

### Limitations

We aim to recruit young people who will go on to have a range of transition experiences and we will analyse any differences in those who do and do not decide to participate.

As in any longitudinal study, there will be a risk of dropout. However, as described earlier, we have incentives in place and the research assistants will receive training guided by recent articles that have examined the factors that make young people more or less likely to remain in a longitudinal study. These include quality of initial visit [92] which incorporates ‘explaining the purpose of the study so that young people are enthused and feel they are making a contribution’, maintaining regular contact between visits [93,94], and giving financial incentives [95]. Initial baseline data will be available for all participants so comparisons can be made between those who continue in the study and those who drop out.

## Abbreviations

ASD: Autism spectrum disorder; CHN: Complex health need; DCE: Discrete choice experiment; NHS: National Health Service; RA: Research assistant.

## Competing interests

The authors declare that they have no competing interests.

## Authors’ contributions

AC, HMcC, TR, LV, ALeC, MP, JP wrote the successful application for funding. AC, HM, HMcC led on the writing of this manuscript and all authors read and approved the final version.

## Pre-publication history

The pre-publication history for this paper can be accessed here:

http://www.biomedcentral.com/1471-2458/13/675/prepub

## Transition Collaborative Group

The Group consists of the authors of this paper and the following collaborators: Dr Angela Bate, Dr Stuart Bennet, Dr Amanda Billson, Dr Stephen Bruce, Dr Gail Dovey-Pearce, Dr Zilla Huma, Dr John Macfarlane, Dr Janet McDonagh, Dr Cara Maiden, Ms Melanie Meek, Dr Jenny Milne, Dr Julie Owens, Dr Jackie Parkes, Ms Debbie Reape, Dr Nandu Thalange, Ms Helen Wheatley; and the following research associates: Kamar Ameen-Ali, Albert Ferrer, Guiomar Garcia-Jalon, Kate Hardenberg, Jenni Hislop, Helen Mason, Sarah McAfferty, Holly Roper, Tracy Scott, Louise Ting, Hazel Windmill, Victoria Wood.
